# Supplementary material for: Assessing species coverage and assembly quality of rapidly accumulating sequenced genomes
Source: Gigascience. 2022 Feb 25;11:giac006. doi: 10.1093/gigascience/giac006 (PMC8881204; doi:10.1093/gigascience/giac006)
Supplement: giac006_Response_to_Reviewer_Comments_Revision_1 [file giac006_response_to_reviewer_comments_revision_1.pdf]

Author's Response To Reviewer Comments

Close

In addition, please register any new software application in the bio.tools and SciCrunch.org databases to receive RRID (Research Resource Identification Initiative ID) and biotoolsID identifiers, and include these in your manuscript. This will facilitate tracking, reproducibility and re-use of your tool.
Response: We have registered the application at the bio.tools and SciCrunch.org databases and obtained a biotools ID (biotools:arthropoda_assembly_assessment_catalogue) as well as a RRID (RRID:SCR_021864).

Reviewer #1: We are now entering a period with rapidly increasing numbers arthropod genome assemblies. Quality has vastly improved because of new high quality long read technologies, but still has a chance to be uneven. Comparative genomics requires at least some effort to ensure the datasets are comparable. Here the authors have produced a nice tool to help find sequenced arthropod genomes and compare their quality. They use their previous experience with BUSCO to measure quality, and overall I expect will be using this resource quite a lot. I also expect a lot of people will use this resource to identify high quality assemblies for comparative analysis.
Response: We thank Reviewer #1 for their positive reception of the workflow and the community resources we have developed.

One possible plot that would be useful would be completeness plots - things like number of orders with a representative, families etc, partly to show progress, and partly so missing taxa can be easily identified.
Response: Figure 1 in the main text is designed to give this overview at the Order level. We have now added a similar overview plot to the Plots page of the website.

The manuscript is well written, but more importantly the data and methods are easily accessed, and everything is well written up. The tool and website does what it says on the tin, and I can't really see any reason not to publish rapidly.
Response: We greatly appreciate the enthusiasm - thank you!

Reviewer #2: The publication describes a useful tool to quickly survey a range of QC metrics for genomes available in NCBI. The a3cat toolkit can be used to setup as well as update the assessment results for public or private assemblies for a user-defined taxon. Overall, the website and the workflow on gitlab are a useful resource for the genomics community ask a number of comparative genomics questions. I enjoyed reading this manuscript and only have minor comments. I would like to bring some more use cases to the attention of the authors that can enrich the discussion. The authors have already presented nuggets from the data mining of results but here are a few thoughts to add to the value of results reported here, as that can be further improved.
Response: We thank Reviewer #2 for their appreciation of the workflow and the community resources we have developed, and we respond accordingly to the suggestions below.

Given an assembly from an insect with an approximate taxonomic classification based on morphology or genetic markers, can the a3cat results be used to figure out the best reference genome or a set of closely related genomes for comparative analysis of the gene space? One idea could be to use the overlap of lineage specific BUSCO genes found in the new genome with BUSCO genes present in other assemblies to identify related genomes.
Response: This is an interesting idea that could certainly be explored with the BUSCO assessment data we have generated and shared through the A3Cat. The resource in its current form is designed to be clean and simple by offering querying and data retrieval rather than data analysis. An analysis platform would require substantial additional development, we therefore offer the data in an easily findable way so that community users can then perform tailored downstream analyses such as overlap analyses suggested here.

The discussion covers results when the results are filtered by level (contig, scaffold, chromosome) or type (haploid, principal or alternate pseudohaplotype). It might be worthwhile to further segment the results based on input raw data (for e.g. short reads, short reads + mate pair, long reads) to explore if the contiguity of the assembly and completeness and duplication of the gene space is impacted by the proportion of indels in the raw reads irrespective of the length of the reads. There a number of other relevant variables like assembly algorithm and parameters but that can lead to very sparse data.
Response: The metadata available from the NCBI describing these additional variables are somewhat inconsistently applied, heterogenous in the labels used, and often missing or unclear, making automated analyses unfeasible. Furthermore, explorations of these factors were recently presented in a snapshot assessment of 601 insect assemblies (Hotaling et al. 2021). We therefore decided to focus on aspects that were readily automatable, in the context of providing an updateable resource. We have now added a specific point to the main text to highlight that additional partitioning can be performed but would normally require metadata curation.

The authors talk about the proportion of repeat content in larger genomes. This might be a valuable resource to add to the a3cat results as initiatives like Ag100Pest and DToL are producing high quality insect genomes >1-2Gbp with a large number of repeats that are going to be better assembled than ever before with high fidelity long reads. Adding the results of a widely used de novo repeat identification tool like RepeatModeler based on the DFAM database will provide a consistent measure of repeat content across all analyzed genomes and add to the value of this toolkit. In case some of this information is already available in NCBI, it can be pulled using the API avoiding the need for this massive compute job.
Response: We certainly consider this as a useful extra attribute to add to the catalogue of assembly metrics, so we had already investigated the current feasibility of retrieving these metadata from the NCBI. For those assemblies with RepeatMasker outputs provided on the NCBI FTP sites it is possible to extract some relevant metadata on repeat content, but this is not trivial. The continued development of the NCBI API tools greatly facilitates database querying and data retrieval, so when repeat content metadata are made accessible through the API we look forward to being able to add these features to future versions of the workflow and catalogue.

This next issue is related to BUSCO but effects the results and conclusions of the a3cat tool. Is it possible that some of the BUSCO marker genes (from OrthoDB9 or 10) are based on short read assemblies with minor errors in gene models? When run on recent assemblies based on high fidelity long reads with the correctly assembled gene model, BUSCO might report the marker as missing or fragmented. I understand this outside the scope of this paper but if this is possible, it should be mentioned as a potential pitfall.
Response: We agree that this is more of a technical discussion on how BUSCO performs and thus out of scope here. We do acknowledge that it is important to mention possible limitations of BUSCO given that the a3cat is presenting BUSCO results. We have therefore added additional information on how to interpret BUSCO results to the a3cat website (About page).

A common problem with bioinformatics resources is the lack of a sustainability plan. I know this is difficult to pin down for the mid or long term in the face of unpredictable funding but I would like to encourage the authors to present a plan to manage and update the web resource if at all possible.
Response: The management and updating of the current resource is relatively lightweight in terms of overheads as the whole workflow is designed to produce easily updatable results that add to the existing results. The workflow implementation and dependencies management makes it easy to run on any other compute platform where future updates (even by other groups) would not require re-running any existing assessments. We would rather not detail specific long-term sustainability plans in the manuscript itself as this implies some sort of obligation, but we are happy to elaborate on alternative solutions already considered: (i) the BUSCO developers themselves (Zdobnov Group, University of Geneva, https://www.ezlab.org/) could take over or provide support to sustain our resource; (ii) the Swiss Institute of Bioinformatics could also take over or provide support to sustain our resource as they do for several other bioinformatics tools and resources (https://www.sib.swiss/research-infrastructure/database-software-tools/sib-resources); or (iii) the arthropod genomics community resource, the i5k Workspace @NAL (https://i5k.nal.usda.gov/) could provide an alternative hosting solution as they are already mandated to serve the needs of the community.

For future work, it might be a good idea to consider the extension of the a3cat toolkit to include other metrics beyond the current contiguity and gene space completeness measures. Mash or ANI distances are becoming computationally tractable for large data sets. I have already mentioned the repeat content issue. Long range similarity measures based on Hi-C data or nucleotide composition based on kmer analysis might be other items to ponder.
Response: Here we have focused on attributes that are (i) straightforward to obtain directly from the NCBI, and (ii) produced as outputs of running BUSCO. We believe that these provide a solid baseline overview that already serves the most pressing needs of the community. We do however agree that adding additional features would enhance the utility of the resource, especially for more specialist users interested in more detailed metrics. In balancing sustainability and updatebility with feature comprehensiveness we have opted for a combination of attributes that is useful but not overly onerous or compute intensive to generate. The workflow of course remains fully extensible, and thus we have now mentioned in the manuscript some of these added extras and possible features for future development.

Minor revisions
Since the logic and applicability of this work is so straightforward, some of the text can be shortened to reduce duplication. For e.g. on Pg 4 this paragraph can be shortened, "Using their Complete Proteome…. for selected groups of species from their field of interest." In the same paragraph, I see "(i) aid project design, particularly in the context of comparative genomics analyses; (ii) simplify comparisons of the quality of their own data with that of existing assemblies; and (iii) provide a means to survey accumulating genomics resources of interest to their ongoing research projects." Can the difference between (i) and (iii) be clearly explained?
Response: We have been through the text and removed or simplified statements that are somewhat repetitive, particularly in the paragraph mentioned. The intended distinction between (i) and (iii) was that (i) refers specifically to deciding which species to include in a given comparative genomics analysis while (iii) refers more generally to simply keeping up-to-date with accumulating resources. We have edited these to improve clarity.

Typographical errors
Response: Thank you for pointing these out, they have now both been corrected.

On Pg 8, the abbreviation CoL- needs an explanation.
On Pg 12, can the term span be elaborated?

Close
