# Supplementary material for: Assessing species coverage and assembly quality of rapidly accumulating sequenced genomes
Source: Gigascience. 2022 Feb 25;11:giac006. doi: 10.1093/gigascience/giac006 (PMC8881204; doi:10.1093/gigascience/giac006)
Supplement: giac006_Reviewer_1_Report_Original_Submission — Stephen Richards -- 10/29/2021 Reviewed [file giac006_reviewer_1_report_original_submission.pdf]

Reviewer Report

Title: Assessing species coverage and assembly quality of rapidly accumulating sequenced genomes

Version: Original Submission **Date:** 10/29/2021

Reviewer name: Stephen Richards

Reviewer Comments to Author:

We are now entering a period with rapidly increasing numbers arthropod genome assemblies.
Quality has vastly improved because of new high quality long read technologies, but still has a chance to be uneven.
Comparative genomics requires at least some effort to ensure the datasets are comparable.
Here the authors have produced a nice tool to help find sequenced arthropod genomes and compare their quality.
They use their previous experience with BUSCO to measure quality, and overall I expect will be using this resource quite a lot.
I also expect a lot of people will use this resource to identify high quality assemblies for comparative analysis.
One possible plot that would be useful would be completeness plots - things like number of orders with a representative, families etc,
partly to show progress, and partly so missing taxa can be easily identified.
The manuscript is well written, but more importantly the data and methods are easily accessed, and everything is well written up.
The tool and website does what it says on the tin, and I can't really see any reason not to publish rapidly.

Level of Interest

Please indicate how interesting you found the manuscript: Choose an item.

Quality of Written English

Please indicate the quality of language in the manuscript: Choose an item.

Declaration of Competing Interests

Please complete a declaration of competing interests, considering the following questions:

- Have you in the past five years received reimbursements, fees, funding, or salary from an organisation that may in any way gain or lose financially from the publication of this manuscript, either now or in the future?

- Do you hold any stocks or shares in an organisation that may in any way gain or lose financially from the publication of this manuscript, either now or in the future?
- Do you hold or are you currently applying for any patents relating to the content of the manuscript?
- Have you received reimbursements, fees, funding, or salary from an organization that holds or has applied for patents relating to the content of the manuscript?
- Do you have any other financial competing interests?
- Do you have any non-financial competing interests in relation to this paper?

If you can answer no to all of the above, write 'I declare that I have no competing interests' below. If your reply is yes to any, please give details below.

I declare that I have no competing interests'

I agree to the open peer review policy of the journal. I understand that my name will be included on my report to the authors and, if the manuscript is accepted for publication, my named report including any attachments I upload will be posted on the website along with the authors' responses. I agree for my report to be made available under an Open Access Creative Commons CC-BY license (http://creativecommons.org/licenses/by/4.0/). I understand that any comments which I do not wish to be included in my named report can be included as confidential comments to the editors, which will not be published.

Choose an item.

To further support our reviewers, we have joined with Publons, where you can gain additional credit to further highlight your hard work (see: https://publons.com/journal/530/gigascience). On publication of this paper, your review will be automatically added to Publons, you can then choose whether or not to claim your Publons credit. I understand this statement.

Yes Choose an item.
