# Supplementary material for: Assessing species coverage and assembly quality of rapidly accumulating sequenced genomes
Source: Gigascience. 2022 Feb 25;11:giac006. doi: 10.1093/gigascience/giac006 (PMC8881204; doi:10.1093/gigascience/giac006)
Supplement: giac006_Reviewer_2_Report_Original_Submission — Surya Saha -- 12/4/2021 Reviewed [file giac006_reviewer_2_report_original_submission.pdf]

Reviewer Report

Title: Assessing species coverage and assembly quality of rapidly accumulating sequenced genomes

Version: Original Submission **Date:** 12/4/2021

Reviewer name: Surya Saha

Reviewer Comments to Author:

The publication describes a useful tool to quickly survey a range of QC metrics for genomes available in NCBI. The a3cat toolkit can be used to setup as well as update the assessment results for public or private assemblies for a user-defined taxon. Overall, the website and the workflow on gitlab are a useful resource for the genomics community ask a number of comparative genomics questions. I enjoyed reading this manuscript and only have minor comments. I would like to bring some more use cases to the attention of the authors that can enrich the discussion.
The authors have already presented nuggets from the data mining of results but here are a few thoughts to add to the value of results reported here, as that can be further improved.
Given an assembly from an insect with an approximate taxonomic classification based on morphology or genetic markers, can the a3cat results be used to figure out the best reference genome or a set of closely related genomes for comparative analysis of the gene space? One idea could be to use the overlap of lineage specific BUSCO genes found in the new genome with BUSCO genes present in other assemblies to identify related genomes.
The discussion covers results when the results are filtered by level (contig, scaffold, chromosome) or type (haploid, principal or alternate pseudohaplotype). It might be worthwhile to further segment the results based on input raw data (for e.g. short reads, short reads + mate pair, long reads) to explore if the contiguity of the assembly and completeness and duplication of the gene space is impacted by the proportion of indels in the raw reads irrespective of the length of the reads. There a number of other relevant variables like assembly algorithm and parameters but that can lead to very sparse data.
The authors talk about the proportion of repeat content in larger genomes. This might be a valuable resource to add to the a3cat results as initiatives like Ag100Pest and DToL are producing high quality insect genomes >1-2Gbp with a large number of repeats that are going to be better assembled than ever before with high fidelity long reads. Adding the results of a widely used de novo repeat identification tool like RepeatModeler based on the DFAM database will provide a consistent measure of repeat content across all analyzed genomes and add to the value of this toolkit. In case some of this information is already available in NCBI, it can be pulled using the API avoiding the need for this massive compute job.
This next issue is related to BUSCO but effects the results and conclusions of the a3cat tool. Is it possible that some of the BUSCO marker genes (from OrthoDB9 or 10) are based on short read assemblies with minor errors in gene models? When run on recent assemblies based on high fidelity long reads with the correctly assembled gene model, BUSCO might report the marker as missing or fragmented. I understand this outside the scope of this paper but if this is possible, it should be mentioned as a potential pitfall.

A common problem with bioinformatics resources is the lack of a sustainability plan. I know this is difficult to pin down for the mid or long term in the face of unpredictable funding but I would like to encourage the authors to present a plan to manage and update the web resource if at all possible.
For future work, it might be a good idea to consider the extension of the a3cat toolkit to include other metrics beyond the current contiguity and gene space completeness measures. Mash or ANI distances are becoming computationally tractable for large data sets. I have already mentioned the repeat content issue. Long range similarity measures based on Hi-C data or nucleotide composition based on kmer analysis might be other items to ponder.
Minor revisions
Since the logic and applicability of this work is so straightforward, some of the text can be shortened to reduce duplication. For e.g. on Pg 4 this paragraph can be shortened, "Using their Complete Proteome…. for selected groups of species from their field of interest." In the same paragraph, I see "(i) aid project design, particularly in the context of comparative genomics analyses; (ii) simplify comparisons of the quality of their own data with that of existing assemblies; and (iii) provide a means to survey accumulating genomics resources of interest to their ongoing research projects." Can the difference between (i) and (iii) be clearly explained?
Typographical errors
On Pg 8, the abbreviation CoL- needs an explanation.
On Pg 12, can the term span be elaborated?

Level of Interest

Please indicate how interesting you found the manuscript: Choose an item.

Quality of Written English

Please indicate the quality of language in the manuscript: Choose an item.

Declaration of Competing Interests

Please complete a declaration of competing interests, considering the following questions:

- Have you in the past five years received reimbursements, fees, funding, or salary from an organisation that may in any way gain or lose financially from the publication of this manuscript, either now or in the future?
- Do you hold any stocks or shares in an organisation that may in any way gain or lose financially from the publication of this manuscript, either now or in the future?
- Do you hold or are you currently applying for any patents relating to the content of the manuscript?
- Have you received reimbursements, fees, funding, or salary from an organization that holds or has applied for patents relating to the content of the manuscript?

- Do you have any other financial competing interests?
- Do you have any non-financial competing interests in relation to this paper?

If you can answer no to all of the above, write 'I declare that I have no competing interests' below. If your reply is yes to any, please give details below.

I declare that I have no competing interests

I agree to the open peer review policy of the journal. I understand that my name will be included on my report to the authors and, if the manuscript is accepted for publication, my named report including any attachments I upload will be posted on the website along with the authors' responses. I agree for my report to be made available under an Open Access Creative Commons CC-BY license (http://creativecommons.org/licenses/by/4.0/). I understand that any comments which I do not wish to be included in my named report can be included as confidential comments to the editors, which will not be published.

Choose an item.

To further support our reviewers, we have joined with Publons, where you can gain additional credit to further highlight your hard work (see: https://publons.com/journal/530/gigascience). On publication of this paper, your review will be automatically added to Publons, you can then choose whether or not to claim your Publons credit. I understand this statement.

Yes Choose an item.
